# *PPARG* rs3856806 C>T Polymorphism Increased the Risk of Colorectal Cancer: A Case-Control Study in Eastern Chinese Han Population

**DOI:** 10.3389/fonc.2019.00063

**Published:** 2019-02-19

**Authors:** Jing Lin, Yu Chen, Wei-feng Tang, Chao Liu, Sheng Zhang, Zeng-qing Guo, Gang Chen, Xiong-wei Zheng

**Affiliations:** ^1^Cancer Bio-immunotherapy Center, Fujian Medical University Cancer Hospital & Fujian Cancer Hospital, Fuzhou, China; ^2^Department of Medical Oncology, Fujian Medical University Cancer Hospital & Fujian Cancer Hospital, Fuzhou, China; ^3^Fujian Provincial Key Laboratory of Translational Cancer Medicine, Fuzhou, China; ^4^Department of Cardiothoracic Surgery, Affiliated People's Hospital of Jiangsu University, Zhenjiang, China; ^5^Department of General Surgery, Changzhou No. 3 People's Hospital, Changzhou, China; ^6^Department of Pathology, Fujian Medical University Cancer Hospital & Fujian Cancer Hospital, Fuzhou, China

**Keywords:** *PPARG*, *PPARGC1A*, *PPARGC1B*, polymorphism, colorectal cancer, risk

## Abstract

**Purpose:** Functional variants in the *peroxisome proliferator-activated receptor gamma* (*PPARG*) and *PPARG co-activator 1* (*PPARGC1*) family (e.g., *PPARGC1A* and *PPARGC1B*) genes were predicted to confer susceptibility to colorectal cancer (CRC). The aim of the present study was to explore the relationship between *PPARG, PPARGC1A, PPARGC1B* polymorphism and the risk of CRC.

**Patients and methods:** We conducted a case-control study with 1,003 CRC cases and 1,303 controls. We selected the *PPARG* rs3856806 C>T, *PPARGC1A* rs2970847 C>T, rs8192678 C>T, rs3736265 G>A and *PPARGC1B* rs7732671 G>C and rs17572019 G>A SNPs to assess the relationship between *PPARG, PPARGC1A, PPARGC1B* their variants and risk of CRC.

**Results:** We found that the *PPARG* rs3856806 C>T polymorphism increased the risk of CRC (TT vs. CC: adjusted OR, 1.59, 95% CI 1.08–2.35, *P* = 0.020; TT/CT vs. CC: adjusted OR, 1.26; 95% CI 1.06–1.49; *P* = 0.009 and TT vs. CC/CT: adjusted OR, 1.54; 95% CI 1.05–2.26; *P* = 0.028), even after a Bonferroni correction test. The stratified analysis revealed that the *PPARG* rs3856806 C>T polymorphism also increased the risk of CRC, especially in male, ≥61 years old, never smoking, never drinking, BMI ≥ 24 kg/m^2^, colon cancer and rectum cancer subgroups.

**Conclusion:** Our findings highlight that the *PPARG* rs3856806 C>T polymorphism may increase the risk of CRC. In the future larger sample size case-control studies with a detailed functional assessment are needed to further determine the relationship of the *PPARG* rs3856806 C>T polymorphism with CRC risk.

## Introduction

Colorectal cancer (CRC) is one of the most common type of malignancies, accounting for 1.8 million cases in GLOBOCAN 2018 (1). The incidence of CRC is increasing in China, where it ranks as the fifth most common carcinoma in male and the fourth in female, with a total of 215,700 patients diagnosed in 2015 (2). Epidemiologic investigations have attributed most of CRC to some important environmental factors (3). The increase of the incidence of CRC is proposed to correlate with an unhealthy lifestyle, including drinking, smoking, low intake of dietary fiber, high intake of dietary fat, decreased consumption of vegetables, and fruits and being physically inactive (4–7). Accumulating evidence highlighted that besides these unhealthy lifestyles and environmental factors, some additional inherited susceptibility factors may be associated with the development of CRC. As CRC is associated with obesity and Waist-to-Hip Ratio (WHR) (8–10), the *peroxisome proliferator-activated receptor gamma* (*PPARG*), *PPARG co-activator 1* (*PPARGC1*) family (e.g., *PPARGC1A* and *PPARGC1B*) may be strong candidate genes predisposing to CRC (11).

*PPARG* is located in 3p25. *PPARG* is also known as *NR1C3 (nuclear receptor subfamily 1, group C, member 3)* which shares some common conservative domains with other steroid receptors (e.g., estrogen, progesterone, retinoid, vitamin D and thyroid receptors). It was reported that *PPARG* is a regulator of adipocyte differentiation, energy homeostasis and obesity (12–14). *PPARG* decreases the inflammatory response of cells (15) and increases synthesis and release of paraoxonase 1 (16). Wang et al. reported that *PPARG* gene might be one of the targets of miRNA-34a and a conceivable therapeutic targets for CRC (17).^.^*PPARGC1A* and *PPARGC1B*, transcriptional co-activators of *PPARG*, may control transcription in adipogenesis, oxidative metabolism genes (18). Thus, *PPARG, PPARGC1A*, and *PPARGC1B* might be implicated in the development of cancer.

Pro12Ala and His449His (rs3856806 C>T) polymorphisms in the *PPARG* gene are two of the most common variants in the *PPARG* gene. Recently, a meta-analysis confirmed that the *PPARG* Pro12Ala polymorphism might decreased the risk of CRC (19). Several case-control studies focused on the potential role of *PPARG* variants in determining CRC susceptibility. The *PPARG* rs3856806 C>T is a common single-nucleotide polymorphism (SNP) in the coding region. Recently, a meta-analysis indicated that the *PPARG* rs3856806 C>T polymorphism may increase the susceptibility of overall cancer (20). In this pooled study, there were seven independent case-control studies with 1,720 cases and 3,458 controls focusing on the association of the *PPARG* rs3856806 C>T polymorphism with CRC risk (21–24). As well, a tendency to increased CRC susceptibility was noted. Because of the lack of sufficient sample sizes, the evidence may be limited. Additionally, (25) reported that the *PPARGC1B* rs7732671 G>C polymorphism may decrease the susceptibility of breast cancer. However, the association between *PPARGC1A* and *PPARGC1B* SNPs and the risk of CRC was unknown. The aim of this case-control study was to assess the association of *PPARG, PPARGC1A*, and *PPARGC1B* polymorphisms with CRC risk. We selected *PPARG* rs3856806 C>T, *PPARGC1A* rs2970847 C>T, rs8192678 C>T, rs3736265 G>A, and *PPARGC1B* rs7732671 G>C and rs17572019 G>A SNPs to determine the relationship between their variants and CRC risk in an Eastern Chinese Han population.

## Materials and Methods

### Study Subjects

This cohort was in part previously studied (19, 26). The CRC cases were recruited from Fujian Medical University Union Hospital (Fuzhou city, China) and the Affiliated People's Hospital of Jiangsu University (Zhenjiang city, China) between October 2014 and August 2017. The major inclusion criteria of CRC cases were: (1) sporadic CRC cases; (2) newly diagnosed CRC patients via pathology; and (3) Han population who living in Eastern China. And the exclusion criteria were: (1) hereditary non-polyposis CRC; (2) CRC cases who have been treated with chemoradiotherapy and (3) with another malignancy history. During the period, a total of 1,186 CRC patients were diagnosed in those local hospitals. Our study includes 1,003 (84.57%) patients, who agree to attend this study and provided blood samples for SNP analysis. The mean age of CRC patients was 61.10 ± 12.17 years. From 1,521 selected controls, 1,303 (85.67%) agreed to participate and donated a biological sample in this study. The controls included 1,303 healthy volunteers who participated in a routine examination in these hospitals, with a mean age of 61.40 ± 9.61 years. For selecting controls, the inclusion criteria were: (1) without a carcinoma history subjects; (2) similar age matched to CRC group; and (3) Han population who is a resident of Eastern China. Additionally, subjects who had a cancer history were excluded. The controls were matched with CRC patients by age and sex. The information on risk factor was obtained from the CRC cases and controls during a medical interview. And weight and height were measured. The body mass index (BMI) was calculated as weight/height^2^ (kg/m^2^) and BMI ≥ 24 kg/m^2^ was considered as overweight and obesity for Chinese (27, 28). All participants enrolled in the present study signed the informed consent and were of Chinese origin. The study protocol was approved by the Ethics Committee of Fujian Medical University and Jiangsu University.

### DNA Extraction and Genotyping

Two milliliters of Ethylenediamine tetra acetic acid (EDTA)-anticoagulated blood was collected from each participant. Blood samples were stored in a −80° C freezer. Using a Promega DNA Blood Mini Kit (Promega, Madison, USA), genomic DNA was isolated from lymphocytes. We placed the cryopreserved specimen at room temperature for an hour. After red blood cell removal, nuclear releasing and protein precipitation, we obtained genomic DNA. We add 300 μl of DNA solution (pH 8.0) and placed the sample in a refrigerator at 4°C for 1–2 weeks. A NanoDrop ND-1000 micro spectrophotometer was used to determine DNA concentration and purity. As described in previous studies, the genotypes of the *PPARG* rs3856806 C>T, *PPARGC1A* rs2970847 C>T, rs8192678 C>T, rs3736265 G>A, and *PPARGC1B* rs7732671 G>C and rs17572019 G>A SNPs was determined by a custom-by-design 48-Plex SNPscan Kit (Genesky Biotechnologies Inc., Shanghai, China) (29, 30). This genotyping method was designed as a multiplex fluorescence PCR (31). Ninety-two DNA samples (4%) were randomly selected and tested by another technician for quality control. The genotypes of these SNPs were not changed.

### Statistical Analysis

We used an online Chi-square software (http://ihg.gsf.de/cgi-bin/hw/hwa1.pl) to test deviation from the Hardy-Weinberg equilibrium (HWE) by using Pearson's goodness-of-fit chi-square. The genotype frequencies of the *PPARG* rs3856806 C>T, *PPARGC1A* rs2970847 C>T, rs8192678 C>T, rs3736265 G>A, and *PPARGC1B* rs7732671 G>C and rs17572019 G>A variants among CRC cases were compared to those of controls using a χ^2^ test or Fisher's exact test. Multivariate logistic regression analysis was harnessed to obtain crude and adjusted odds ratios (ORs) with their 95% confidence intervals (CIs) to predict the relationship of the *PPARG* rs3856806 C>T, *PPARGC1A* rs2970847 C>T, rs8192678 C>T, rs3736265 G>A, and *PPARGC1B* rs7732671 G>C and rs17572019 G>A polymorphisms with susceptibility to CRC. Dominant, recessive, heterozygote and homozygote models were used to evaluate the association of these SNPs with CRC risk. The χ^2^ test or Fisher's exact test was first applied to compare the distribution of age, sex, alcohol consumption, smoking status, and BMI between CRC patients and controls. A *P* < 0.05 (two–tailed) was defined as a significant association. All data were analyzed by SAS software for Windows (9.4 version, SAS Institute, Cary, USA). In this case-control study, a Bonferroni correction test was applied for multiple testing (32, 33). An internal validation the through bootstrap method was applied to *PPARG* rs3856806 C>T. We used 0.623 bootstrap method to resample 1,003 cases from the CRC patient group and 1,303 cases from the control group to validate our results.

## Results

### Study Characteristics

Selected demographic variables and risk factors in the enrolled population and the correlation with CRC are summarized in Table 1. There was no significant difference between CRC patients and controls regarding sex (*P* = 0.867), age (61.10 ± 12.17 years for cases and 61.40 ± 9.61 years for controls, *P* = 0.496), suggesting that these variables were well-matched. Alcohol consumption, BMI and smoking status were statistically different (*P* < 0.001, *P* < 0.001, and *P* = 0.002, respectively) between two groups. The primary information of *PPARG, PPARGC1A*, and *PPARGC1B* SNPs is displayed in Table 2. The genotype distributions of *PPARG* rs3856806 C>T, *PPARGC1A* rs2970847 C>T, rs8192678 C>T, rs3736265 G>A, and *PPARGC1B* rs7732671 G>C and rs17572019 G>A are in accordance with HWE in controls (*P* = 0.143, 0.925, 0.800, 0.059, 0.970, and 0.372, respectively).

**Table 1:** Distribution of selected characteristics in CRC cases and controls.

**Variable**	**Cases (*n* = 1,003)**		**Controls (*n* = 1,303)**		***P*^a^**
	***n***	**%**	***n***	**%**	
Age (years)	62, IQR (53–70)		61, IQR (55–68)		
Age (years)					0.605
< 61	451	44.97	600	46.05	
≥61	552	55.03	703	53.95	
**Sex**					0.867
Male	620	61.81	801	61.47	
Female	383	38.19	502	38.53	
**Smoking status**					**0.002**
Never	744	74.18	1038	79.66	
Ever	259	25.82	265	20.34	
**Alcohol use**					** < 0.001**
Never	829	82.65	1,167	89.56	
Ever	174	17.35	136	10.44	
**BMI (kg/m**^**2**^**)**					
< 24	670	66.80	688	52.80	** < 0.001**
≥ 24	333	33.20	615	47.20	
**Site of tumor**					
Colon cancer	431	42.97			
Rectum cancer	572	57.03			

a *Two-sided χ^2^ test and student t-test*.

*IQR: interquartile range*.

*Bold values are statistically significant (P < 0.05)*.

*BMI: body mass index*.

**Table 2:** Primary information for *PPARG* rs3856806 C>T, *PPARGC1A* rs2970847 C>T, rs8192678 C>T, rs3736265 G>A, and *PPARGC1B* rs7732671 G>C and rs17572019 G>A polymorphisms.

**Genotyped SNPs**	***PPARG* rs3856806 C>T**	***PPARGC1A* rs2970847 C>T**	***PPARGC1A* rs3736265 G>A**	***PPARGC1A* rs8192678 C>T**	***PPARGC1B* rs7732671 G>C**	***PPARGC1B* rs17572019 G>A**
Chromosome	3	4	4	4	5	5
Function	coding-synonymous	coding-synonymous	missense	missense	missense	missense
Chr Pos (NCBI Build 38)	12434058	23814301	23813084	23814039	149832680	149832908
MAF^a^ for Chinese in database^b^	0.25	0.28	0.23	0.35	0.09	0.07
MAF in our controls (*n* = 1,303)	0.22	0.22	0.15	0.44	0.06	0.06
*P* value for HWE^c^ test in our controls	0.143	0.925	0.059	0.800	0.970	0.372
Genotyping method	SNPscan	SNPscan	SNPscan	SNPscan	SNPscan	SNPscan
% Genotyping value	98.87%	98.87%	98.66%	98.87%	98.87%	98.87%

a *MAF: minor allele frequency*.

b *http://gvs.gs.washington.edu/GVS147/*.

c *HWE: Hardy–Weinberg equilibrium*.

### Association of *PPARG* rs3856806 C>T, *PPARGC1A* rs2970847 C>T, rs8192678 C>T, rs3736265 G>A, and *PPARGC1B* rs7732671 G>C and rs17572019 G> A Polymorphisms With CRC Risk

Table 3 summarizes the genotype distributions of *PPARG* rs3856806 C>T, *PPARGC1A* rs2970847 C>T, rs8192678 C>T, rs3736265 G>A, and *PPARGC1B* rs7732671 G>C and rs17572019 G>A SNPs in CRC cases and controls. The genotype frequencies of *PPARG* rs3856806 C>T were 55.51% (CC), 38.16% (CT), and 6.33% (TT) in CRC cases and 60.69% (CC), 35.31% (CT), and 4.00% (TT) in controls. When the frequency of *PPARG* rs3856806 CC genotype was used as a reference, individuals carrying the *PPARG* rs3856806 TT genotype had an increased risk to CRC (crude OR = 1.67, 95% CI 1.13–2.45 for TT vs. CC, *P* = 0.009). When compared with the frequency of *PPARG* rs3856806 CC genotype, individuals carrying the *PPARG* rs3856806 TT/CT genotype also had an increased the risk of CRC (crude OR = 1.24, 95% CI 1.05–1.46 for TT/CT vs. CC, *P* = 0.013). When the frequency of the *PPARG* rs3856806 CC/CT genotype was used as a reference, individuals carrying the *PPARG* rs3856806 TT genotype had a significantly increased susceptibility to CRC (crude OR = 1.62, 95% CI 1.11–2.37 for TT vs. CC/CT, *P* = 0.012). After adjustments for age, sex, smoking, BMI, and drinking, the observed increased susceptibility of CRC was not essentially altered (TT vs. CC: adjusted OR, 1.59, 95% CI 1.08–2.35, *P* = 0.020; TT/CT vs. CC: adjusted OR, 1.26; 95% CI 95% CI 1.06–1.49; *P* = 0.009 and TT vs. CC/CT: adjusted OR, 1.54; 95% CI 95% CI 1.05–2.26; *P* = 0.028), Table 3.

**Table 3:** Logistic regression analyses of associations between *PPARG* rs3856806 C>T, *PPARGC1A* rs2970847 C>T, rs8192678 C>T, rs3736265 G>A, and *PPARGC1B* rs7732671 G>C and rs17572019 G>A polymorphisms and risk of CRC.

**Genotype**	**Cases (*n* = 1,003)**		**Controls (*n* = 1,303)**		**Crude OR (95%CI)**	***P***	**Adjusted OR^**a**^ (95%CI)**	***P***
	***n***	**%**	***n***	**%**				
***PPARG*** **rs3856806 C>T**								
CC	544	55.51	789	60.69	1.00		1.00	
CT	374	38.16	459	35.31	1.14(0.96–1.35)	0.145	1.16(0.97-1.39)	0.095
TT	62	6.33	52	4.00	**1.67(1.13–2.45)**	**0.009**	**1.59(1.08–2.35)**	**0.020**
CT+TT	436	44.49	511	39.31	**1.24(1.05–1.46)**	**0.013**	**1.26(1.06–1.49)**	**0.009**
CC+CT	918	93.67	1,248	96.00	1.00		1.00	
TT	62	6.33	52	4.00	**1.62(1.11–2.37)**	**0.012**	**1.54(1.05–2.26)**	**0.028**
T allele	498	25.41	563	21.65				
***PPARGC1A*** **rs2970847 C>T**								
CC	593	60.51	788	60.62	1.00		1.00	
CT	344	35.10	449	34.54	0.98(0.83–1.17)	0.855	0.97(0.81–1.16)	0.743
TT	43	4.39	63	4.85	0.88(0.59–1.31)	0.520	0.92(0.61–1.38)	0.673
CT+TT	387	39.49	512	39.38	1.00(0.85–1.19)	0.959	1.00 (0.84–1.19)	0.985
CC+CT	937	95.61	1,237	95.15	1.00		1.00	
TT	43	4.39	63	4.85	0.90(0.61–1.34)	0.610	0.95(0.63–1.42)	0.787
T allele	430	21.94	575	22.12				
***PPARGC1A*** **rs3736265 G>A**								
GG	685	70.11	936	72.11	1.00			
GA	260	26.61	322	24.81	1.07(0.88–1.29)	0.493	1.06(0.87–1.29)	0.550
AA	32	3.28	40	3.08	1.06(0.66–1.70)	0.813	1.04(0.64–1.68)	0.885
GA + AA	292	29.89	362	27.89	1.10(0.92–1.32)	0.297	1.09(0.91–1.31)	0.357
GG+GA	945	96.72	1,258	96.92	1.00		1.00	
AA	32	3.28	40	3.08	1.07(0.66–1.71)	0.793	1.05(0.65–1.69)	0.854
A allele	324	16.58	402	15.49				
***PPARGC1A*** **rs8192678 C>T**								
CC	344	35.10	408	31.38	1.00		1.00	
CT	454	46.33	645	49.62	**0.79(0.66–0.95)**	**0.012**	**0.82(0.68–0.98)**	**0.033**
TT	182	18.57	247	19.00	0.83(0.65–1.05)	0.113	0.85(0.66–1.08)	0.171
CT+TT	636	64.90	892	68.62	0.85(0.71–1.01)	0.062	0.87(0.73–1.05)	0.139
CC+CT	798	81.43	1,053	81.00	1.00		1.00	
TT	182	18.57	247	19.00	0.97(0.79–1.20)	0.796	0.98(0.79–1.21)	0.832
T allele	818	41.73	1,139	43.81				
***PPARGC1B*** **rs7732671 G>C**								
GG	863	88.06	1,145	88.08	1.00		1.00	
GC	113	11.53	150	11.54	0.98(0.75–1.27)	0.855	0.99(0.76–1.29)	0.924
CC	4	0.41	5	0.38	1.04(0.28–3.87)	0.957	1.03(0.27–3.88)	0.967
GC+CC	117	11.94	155	11.92	1.00(0.78–1.29)	0.991	1.01(0.78–1.31)	0.927
GG+GC	976	99.59	1,295	99.62	1.00		1.00	
CC	4	0.41	5	0.38	1.06(0.28–3.96)	0.929	1.05(0.28–3.96)	0.946
C allele	121	6.17	160	6.15				
***PPARGC1B*** **rs17572019 G>A**								
GG	862	87.96	1,144	88.00	1.00			
GA	115	11.73	149	11.46	1.00(0.77–1.30)	0.998	1.02(0.79–1.33)	0.877
AA	3	0.31	7	0.54	0.56(0.14–2.15)	0.395	0.47(0.12–1.84)	0.276
GA+AA	118	12.04	156	12.00	1.00(0.78–1.30)	0.976	1.02(0.78–1.32)	0.900
GG+GA	977	99.69	1,293	99.46	1.00		1.00	
AA	3	0.31	7	0.54	0.57(0.15–2.20)	0.412	0.48(0.12–1.86)	0.286
A allele	121	6.17	163	6.27				

a *Adjusted for age, sex, smoking status, alcohol use and BMI status*.

*Bold values are statistically significant (P < 0.05)*.

Table S1 shows the internal validation results through the bootstrap method. When compared with the *PPARG* rs3856806 CC genotype, the *PPARG* rs3856806 TT, and TT/CT genotypes also indicate an increased CRC risk (crude OR = 1.56, 95% CI 1.09–2.23 for TT vs. CC, *P* = 0.015; crude OR = 1.20, 95% CI 1.02–1.42 for TT/CT vs. CC, *P* = 0.033). When compared with the *PPARG* rs3856806 CC/CT genotype, *PPARG* rs3856806 TT genotype also suggest an increased CRC risk (crude OR = 1.53, 95% CI 1.08–2.18 for TT vs. CC/CT, *P* = 0.017). After being adjusted by age, sex, smoking BMI, and drinking, the increased susceptibility of CRC was not essentially altered.

The genotype frequencies of *PPARGC1A* rs8192678 C>T were 35.10% (CC), 46.33% (CT), and 18.57% (TT) in CRC patients and 31.38% (CC), 49.62% (CT), and 19.00% (TT) in healthy controls. When the frequency of the *PPARGC1A* rs8192678 CC genotype was used as a reference, individuals carrying the *PPARGC1A* rs8192678 CT genotype had a decreased susceptibility to CRC (crude OR = 0.79, 95% CI 0.66–0.95 for CT vs. CC, *P* = 0.012). After adjustments for age, sex, smoking, BMI and drinking, this association was also found (CT vs. CC: adjusted OR, 0.82; 95% CI 95% CI 0.68–0.989; *P* = 0.033), Table 3.

We found no significant difference in the genotype distribution of the *PPARGC1A* rs3736265 G>A, rs2970847 C>T and *PPARGC1B* rs7732671 G>C, rs17572019 G>A polymorphisms among CRC cases and controls, Table 3.

The Bonferroni correction test was applied to determine whether the association of the *PPARG* rs3856806 C>T and rs8192678 C>T polymorphisms with the risk of CRC was reliable. We defined the statistical significance level at 0.0125 (0.05/4 genetic models). We found the genotype distribution of that the *PPARG* rs3856806 C>T polymorphism was still significantly different between CRC patients and controls (TT/CT vs. CC: adjusted OR, 1.26; 95% CI 95% CI 1.06–1.49; *P* = 0.009).

### Association of *PPARG* rs3856806 C>T Polymorphism With CRC Risk in a Stratified Analysis

To further assess the association of the *PPARG* rs3856806 C>T polymorphism with CRC risk, we conducted a stratified analysis by BMI, gender, age, tobacco using and alcohol consumption. Table 4 presents the different genotype frequencies of the *PPARG* rs3856806 C>T polymorphism in a subgroup analysis. After an adjustment by logistic regression analysis with gender, age, BMI, tobacco using and drinking status, we found that the *PPARG* rs3856806 C>T polymorphism significantly increased the risk of CRC in several subgroups:1) male subgroup, TT vs. CC, adjusted OR = 1.88, 95% CI 1.14–3.10, *P* = 0.014 and TT vs. CT/CC, adjusted OR = 1.84, 95% CI 1.12–3.02, *P* = 0.016; 2) ≥61 years subgroup, CT/TT vs. CC, adjusted OR = 1.36, 95% CI 1.08–1.71, *P* = 0.010; 3) never smoking subgroup, CT/TT vs. CC, adjusted OR = 1.27, 95% CI 1.05–1.55, *P* = 0.015; 4) never drinking subgroup, CT/TT vs. CC, adjusted OR = 1.27, 95% CI 1.06–1.53, *P* = 0.011; 5) BMI ≥ 24 kg/m^2^ subgroup, TT vs. CC: adjusted OR = 2.65, 95% CI 1.36–5.17, *P* = 0.004; CT/TT vs. CC, adjusted OR = 1.38, 95% CI 1.05–1.81, *P* = 0.022, and TT vs. CT/CC, adjusted OR = 2.51, 95% CI 1.03–4.86, *P* = 0.006 (Table 4).

**Table 4:** Stratified analyses between *PPARG* rs3856806 C>T polymorphism and CRC risk by sex, age, BMI, smoking status, and alcohol consumption.

**Variable**	***PPARG*** **rs3856806 C>T (case/control)^a^**			**Adjusted OR^b^ (95% CI);** ***P***				
	**CC**	**CT**	**TT**	**CC**	**CT**	**TT**	**CT /TT**	**TT vs. (CT/CC)**
**SEX**								
Male	188/382	84/183	15/22	1.00	1.13(0.90–1.41); *P*: 0.296	**1.88(1.14–3.10);** ***P*****: 0.014**	1.25(1.01–1.56); *P*: 0.042	**1.84(1.12–3.02);** ***P*****: 0.016**
Female	146/288	79/135	9/19	1.00	1.23(0.92–1.64); *P*: 0.167	1.25(0.66–2.37); *P*: 0.487	1.26(0.96–1.67); *P*: 0.101	1.18(0.63–2.21); *P*: 0.603
**AGE**								
< 61	155/309	71/152	12/14	1.00	1.06(0.81–1.38); *P*: 0.692	1.77(0.98–3.21); *P*: 0.060	1.15(0.89–1.49); *P*: 0.285	1.76(0.98–3.16); *P*: 0.060
≥61	179/361	92/166	12/27	1.00	1.27(1.00–1.61); *P*: 0.053	1.49(0.88–2.50); *P*: 0.135	**1.36 (1.08–1.71);** ***P*****: 0.010**	1.40(0.84–2.33); *P*: 0.202
**SMOKING STATUS**								
Never	201/541	103/252	13/34	1.00	1.20(0.98–1.47); *P*: 0.078	1.48(0.95–2.30); *P*: 0.082	**1.27(1.05–1.55);** ***P*****: 0.015**	1.41(0.91–2.17); *P*: 0.123
Ever	133/129	60/66	11/7	1.00	1.03(0.71–1.48); *P*: 0.892	2.09(0.87–5.05); *P*: 0.100	1.17(0.82–1.67); *P*: 0.391	2.13(0.89–5.09); *P*: 0.088
**ALCOHOL CONSUMPTION**								
Never	283/623	139/287	22/38	1.00	1.20(0.99–1.45); *P*: 0.067	1.48(0.97–2.26); *P*: 0.072	**1.27(1.06–1.53);** ***P*****: 0.011**	1.41(0.93–2.15); *P*: 0.108
Ever	51/47	24/31	2/3	1.00	1.01(0.62–1.65); *P*: 0.969	2.59(0.89–7.54); *P*: 0.082	1.20(0.75–1.92); *P*: 0.445	2.62(0.91–7.52); *P*: 0.073
**BMI (kg/m**^**2**^**)**								
< 24	210/353	107/171	20/22	1.00	1.13(0.90–1.42); *P*: 0.296	1.26(0.78–2.03); *P*: 0.343	1.18(0.95–1.47); *P*: 0.131	1.23(0.77–1.96); *P*: 0.391
≥24	124/317	56/147	4/19	1.00	1.21(0.92–1.61); *P*: 0.177	**2.65(1.36–5.17);** ***P*****: 0.004**	**1.38(1.05–1.81);** ***P*****: 0.022**	**2.51(1.03–4.86);** ***P*****: 0.006**

a *For PPARG rs3856806 C>T, the genotyping was successful in 980 (97.71%) CRC cases, and 1300 (99.77%) controls*.

b *Adjusted for multiple comparisons in a logistic regression model (age stratified analysis: sex, BMI, smoking status and alcohol consumption adjusted; sex stratified analysis: age, BMI, smoking status and alcohol consumption adjusted; BMI stratified analysis: age, sex, smoking status and alcohol consumption adjusted; smoking stratified analysis: age, sex, BMI and alcohol consumption adjusted and drinking stratified analysis: age, sex, BMI and smoking status adjusted)*.

*Bold values are statistically significant (P < 0.05)*.

### Association of *PPARG* rs3856806 C>T Polymorphism With CRC in a Stratification Group by Site of Tumor

To determine whether the association between the *PPARG* rs3856806 C>T polymorphism and CRC risk was modified by the site of CRC, we conducted stratified analyses. The results of the stratified analyses suggested this SNP increased the risk of colon cancer (CT vs. CC: adjusted OR = 1.27, 95% CI 1.01–1.60, *P* = 0.044 and TT/CT vs. CC: adjusted OR = 1.34, 95% CI 1.07–1.68, *P* = 0.011) and rectum cancer (TT vs. CC: adjusted OR = 1.58, 95% CI 1.01–2.49, *P* = 0.045 and TT vs. CC/CT: adjusted OR = 1.58, 95% CI 1.01–2.46, *P* = 0.043), Table 5.

**Table 5:** Stratified analyses between *PPARG* rs3856806 C>T polymorphism and CRC risk by site of tumor.

**Genotype**	**Controls (*n* = 1,303)**		**Colon cancer cases (*n* = 431)**		**Crude OR (95%CI)**	***P***	**Adjusted OR^a^ (95%CI)**	***P*^a^**	**Rectum cancer cases (*n =* 572)**		**Crude OR (95%CI)**	***P***	**Adjusted OR^a^ (95%CI)**	***P*^a^**
	***n***	**%**	***n***	**%**					***n***	**%**				
***PPARG*** **rs3856806 C>T**														
CC	789	60.69	228	53.90	1.00		1.00		316	56.72	1.00		1.00	
CT	459	35.31	170	40.19	1.24(0.99–1.56)	0.062	**1.27(1.01–1.60)**	**0.044**	204	36.62	1.06(0.86–1.31)	0.564	1.08(0.88–1.34)	0.459
TT	52	4.00	25	5.91	1.61(0.98–2.66)	0.060	1.54(0.93–2.55)	0.093	37	6.64	**1.70(1.10–2.65)**	**0.018**	**1.58(1.01–2.49)**	**0.045**
CT+TT	511	39.31	195	46.10	**1.32(1.06–1.65)**	**0.014**	**1.34(1.07–1.68)**	**0.011**	241	43.27	1.18(0.96–1.44)	0.111	1.19(0.97–1.46)	0.099
CC+CT	1,248	96.00	398	94.09	1.00		1.00		520	93.36	1.00		1.00	
TT	52	4.00	25	5.91	1.51(0.92–2.46)	0.101	1.43(0.87–2.34)	0.160	37	6.64	**1.71(1.11–2.64)**	**0.016**	**1.58(1.01–2.46)**	**0.043**
T allele	563	21.65	220	26.00					278	24.96				

a *Adjusted for age, sex, smoking status, alcohol use and BMI status*.

*Bold values are statistically significant (P < 0.05)*.

## Discussion

Accumulating evidence has highlighted that CRC is associated with obesity and Waist-to-Hip Ratio (WHR) (8–10). Some important metabolism-related genes may be strong candidates for predisposing to CRC (11). *PPARG* may be implicated in metabolism, inflammatory response, adipose cell differentiation, and cellular apoptosis (34–37). The *PPARGC1* family (e.g., *PPARGC1A, PPARGC1B*) also regulate fatty acid oxidation, gluconeogenesis and adaptive thermogenesis (38). These proteins may be involved in the development of obesity. Several studies have focused on the association between the *PPARG* rs3856806 C>T polymorphism and the risk of CRC (21–24). However, the results were inconsistent. In addition, the potential relationships of the *PPARGC1A, PPARGC1B* SNPs with the development of CRC are unknown. To shed some light on this issue, we carried out a case-control study in Eastern Chinese Han population. Our findings suggested that the *PPARG* rs3856806 C>T polymorphism is associated with an increased risk of CRC, especially in male, ≥ 61 years old, never smoking, never drinking, BMI ≥24 kg/m^2^, colon cancer, and rectum cancer subgroups.

*PPARG* is one of the three subtypes of *peroxisome proliferator-activated receptors (PPARs)*. The *PPARG* gene encodes a member of the *PPAR* subfamily of nuclear receptors, which form heterodimers with *retinoid X receptors (RXRs)* and then influence the transcription of many target genes. A previous study concluded that there was evidence for a relationship between obesity and overweight with a risk of colon and rectum cancer (39). A common functional polymorphism (His449His; rs3856806) in *PPARG* is a C → T coding-synonymous substitution in codon 449 of exon 6. Grygiel-Górniak and colleagues reported that higher BMI and visceral fat deposition were promoted by the presence of the *PPARG* rs3856806 T allele (40). Previous studies suggested a potential correlation of this SNP with atherosclerosis, type 2 diabetes and cancer (20, 41–44). Although rs3856806 is a coding-synonymous SNP, it is proposed that a C → T substitution could alter the expression of *PPARG* protein by altering mRNA processing or translation. Doecke et al. reported that the *PPARG* rs3856806 CT genotype may increase the susceptibility of adenocarcinoma of the esophagus in an obesity subgroup (BMI ≥ 30 kg/m^2^) (45). The *PPARG* rs3856806 C>T polymorphism was also found to be significantly over-represented in sporadic glioblastoma multiforme in American populations (46). Jiang et al. reported that the *PPARG* rs3856806 C>T polymorphism was associated with an increased risk of CRC in India (21). However, other case-control studies suggested that *PPARG* rs3856806 C>T might not influence the development of CRC (22–24). Thus, the results were inconsistent and ambiguous. Considering a common SNP having low penetrance susceptibility to cancer, we performed a case-control study with large sample sizes to obtain a more precise assessment. As demonstrated in the results, we found that the *PPARG* rs3856806 C>T polymorphism was associated with an increased risk of CRC, even after a Bonferroni correction test. Thus, our findings were reliable. Recently, a meta-analysis reported that the *PPARG* rs3856806 C>T polymorphism increased the risk of overall cancer (20). Our findings were very similar to this pooled-analysis. Additionally, it is worth noting that we found the that the *PPARG* rs3856806 C>T polymorphism was associated with an increased risk of CRC in the BMI ≥ 24 kg/m^2^ subgroup. It suggested that this SNP might be implicated in the development of obesity and overweight, and subsequently lead to an increased risk to CRC.

There are, however, several limitations in this case-control study. First, the CRC patients and non-cancer controls were from two local hospitals. The potential selection bias might have occurred. Second, a replicated study focusing on the association of these SNPs with CRC risk was not carried out. Third, although we took some risk factors into consideration such as BMI, gender, age, drinking, and smoking status, many other environmental and lifestyle factors, possibly related to the development of CRC, were not collected in this study. Fourth, due to the moderate sample size in some subgroups, the power might be limited. Fifth, a functional study for the *PPARG* rs3856806 C>T polymorphism has not been conducted. Finally, in the future, it is necessary to carry out a functional study to identify the mechanism of the *PPARG* rs3856806 C>T polymorphism.

In conclusion, our findings suggest that the *PPARG* rs3856806 C>T polymorphism may increase the risk of CRC. In the future, larger sample size case-control studies with a detailed functional assessment are needed to further evaluate the relationship of *PPARG* rs3856806 C>T polymorphism with CRC risk.

## Author Contributions

JL, YC, GC, and XZ conceived and designed the experiments. YC, WT, CL, and GC performed the experiments. JL, YC, SZ, and ZG analyzed the data. JL, YC, and XZ contributed reagents, materials, and analysis tools. JL, YC, and WT wrote the paper.

### Conflict of Interest Statement

The authors declare that the research was conducted in the absence of any commercial or financial relationships that could be construed as a potential conflict of interest.

## References

[1] Bray F Ferlay J Soerjomataram I Siegel RL Torre LA Jemal A. Global cancer statistics 2018: GLOBOCAN estimates of incidence and mortality worldwide for 36 cancers in 185 countries. CA Cancer J Clin. (2018) 68:394–424. 10.3322/caac.21492 30207593

[2] Chen W Zheng R Baade PD Zhang S Zeng H Bray F. Cancer statistics in China, 2015. CA Cancer J Clin. (2016) 66:115–32. 10.3322/caac.21338 26808342

[3] Jemal A Bray F Center MM Ferlay J Ward E Forman D. Global cancer statistics. CA Cancer J Clin. (2011) 61:69–90. 10.3322/caac.20107 21296855

[4] Weitz J Koch M Debus J Hohler T Galle PR Buchler MW. Colorectal cancer. Lancet (2005) 365:153–65. 10.1016/S0140-6736(05)17706-X 15639298

[5] Gerber M. Background review paper on total fat, fatty acid intake and cancers. Ann Nutr Metabol. (2009) 55:140–61. 10.1159/000229000 19752540

[6] Xu M Chen YM Huang J Fang YJ Huang WQ Yan B. Flavonoid intake from vegetables and fruits is inversely associated with colorectal cancer risk: a case-control study in China. Br J Nutr. (2016) 116:1275–87. 10.1017/S0007114516003196 27650133

[7] Nagle CM Wilson LF Hughes MC Ibiebele TI Miura K Bain CJ. Cancers in Australia in 2010 attributable to inadequate consumption of fruit, non-starchy vegetables and dietary fibre. Aust N Zealand J Public Health (2015) 39:422–8. 10.1111/1753-6405.12449 26437726 PMC4606769

[8] Dong Y Zhou J Zhu Y Luo L He T Hu H. Abdominal obesity and colorectal cancer risk: systematic review and meta-analysis of prospective studies. Biosci Rep. (2017) 37:BSR20170945. 10.1042/BSR20170945 29026008 PMC5725611

[9] Shirakami Y Ohnishi M Sakai H Tanaka T Shimizu M. Prevention of colorectal cancer by targeting obesity-related disorders and inflammation. Intl J Mol Sci. (2017) 18:E908. 10.3390/ijms18050908 28445390 PMC5454821

[10] Lund EK Belshaw NJ Elliott GO Johnson IT. Recent advances in understanding the role of diet and obesity in the development of colorectal cancer. Proc Nutr Soc. (2011) 70:194–204. 10.1017/S0029665111000073 21385524

[11] Motawi TK Shaker OG Ismail MF Sayed NH. Peroxisome proliferator-activated receptor gamma in obesity and colorectal cancer: the role of epigenetics. Sci Reports (2017) 7:10714. 10.1038/s41598-017-11180-6 28878369 PMC5587696

[12] Guazzoni G Montorsi F Colombo R Di Girolamo V Da Pozzo L Rigatti P. Long term experience with the prostatic spiral for urinary retention due to benign prostatic hyperplasia. Scand J Urol Nephrol. (1991) 25:21–4. 10.3109/00365599109024523 1710824

[13] AlSaleh A Sanders TA O'Dell SD. Effect of interaction between PPARG, PPARA and ADIPOQ gene variants and dietary fatty acids on plasma lipid profile and adiponectin concentration in a large intervention study. Proc Nutr Soc. (2012) 71:141–53. 10.1017/S0029665111003181 22040870

[14] Barbieri M Rizzo MR Papa M Acampora R De Angelis L Olivieri F. Role of interaction between variants in the PPARG and interleukin-6 genes on obesity related metabolic risk factors. Exp Gerontol. (2005) 40:599–604. 10.1016/j.exger.2005.05.004 16029943

[15] Hamblin M Chang L Fan Y Zhang J Chen YE. PPARs and the cardiovascular system. Antioxidants Redox Signal. (2009) 11:1415–52. 10.1089/ars.2008.2280 19061437 PMC2737093

[16] Khateeb J Gantman A Kreitenberg AJ Aviram M Fuhrman B. Paraoxonase 1 (PON1) expression in hepatocytes is upregulated by pomegranate polyphenols: a role for PPAR-gamma pathway. Atherosclerosis (2010) 208:119–25. 10.1016/j.atherosclerosis.2009.08.051 19783251

[17] Wang T Xu H Liu X Chen S Zhou Y Zhang X. Identification of Key Genes in Colorectal Cancer Regulated by miR-34a. Med Sci Monitor (2017) 23:5735–43. 10.12659/MSM.904937 29197895 PMC5724350

[18] Puigserver P Spiegelman BM. Peroxisome proliferator-activated receptor-gamma coactivator 1 alpha (PGC-1 alpha): transcriptional coactivator and metabolic regulator. Endocr Rev. (2003) 24:78–90. 10.1210/er.2002-0012 12588810

[19] Jiang J Xie Z Guo J Wang Y Liu C Zhang S. Association of PPARG rs 1801282 C>G polymorphism with risk of colorectal cancer: from a case-control study to a meta-analysis. Oncotarget (2017) 8:100558–69. 10.18632/oncotarget.20138 29246001 PMC5725043

[20] Ding H Chen Y Qiu H Liu C Wang Y Kang M. PPARG c.1347C>T polymorphism is associated with cancer susceptibility: from a case-control study to a meta-analysis. Oncotarget (2017) 8:102277–90. 10.18632/oncotarget.20925 29254243 PMC5731953

[21] Jiang J Gajalakshmi V Wang J Kuriki K Suzuki S Nakamura S Influence of the C161T but not Pro12Ala polymorphism in the peroxisome proliferator-activated receptor-gamma on colorectal cancer in an Indian population. Cancer Sci. (2005) 96:507–12. 10.1111/j.1349-7006.2005.00072.x 16108832 PMC11160068

[22] Vogel U Christensen J Dybdahl M Friis S Hansen RD Wallin H. Prospective study of interaction between alcohol, NSAID use and polymorphisms in genes involved in the inflammatory response in relation to risk of colorectal cancer. Mutation Res. (2007) 624:88–100. 10.1016/j.mrfmmm.2007.04.006 17544013

[23] Siezen CL Bueno-de-Mesquita HB Peeters PH Kram NR van Doeselaar M van Kranen HJ. Polymorphisms in the genes involved in the arachidonic acid-pathway, fish consumption and the risk of colorectal cancer. Int J Cancer (2006) 119:297–303. 10.1002/ijc.21858 16482563

[24] Kuriki K Hirose K Matsuo K Wakai K Ito H Kanemitsu Y. Meat, milk, saturated fatty acids, the Pro12Ala and C161T polymorphisms of the PPARgamma gene and colorectal cancer risk in Japanese. Cancer Sci. (2006) 97:1226–35. 10.1111/j.1349-7006.2006.00314.x 16965392 PMC11160037

[25] Martinez-Nava GA Burguete-Garcia AI Lopez-Carrillo L Hernandez-Ramirez RU Madrid-Marina V Cebrian ME. PPARgamma and PPARGC1B polymorphisms modify the association between phthalate metabolites and breast cancer risk. Biomarkers (2013) 18:493–501. 10.3109/1354750X.2013.816776 23866026

[26] Zhang S Chen S Chen Y Kang M Liu C Qiu H. Investigation of methylenetetrahydrofolate reductase tagging polymorphisms with colorectal cancer in Chinese Han population. Oncotarget (2017) 8:63518–27. 10.18632/oncotarget.18845 28969008 PMC5609940

[27] Zhai Y Zhao WH Chen CM. [Verification on the cut-offs of waist circumference for defining central obesity in Chinese elderly and tall adults]. Zhonghua Liu Xing Bing Xue Za Zhi. (2010) 31:621–5. 21163090

[28] Zhang X Zhang S Li Y Detrano RC Chen K Li X. Association of obesity and atrial fibrillation among middle-aged and elderly Chinese. Int J Obes. (2009) 33:1318–25. 10.1038/ijo.2009.157 19668255

[29] Chen X Li S Yang Y Yang X Liu Y Liu Y. Genome-wide association study validation identifies novel loci for atherosclerotic cardiovascular disease. J Thromb Haemost. (2012) 10:1508–14. 10.1111/j.1538-7836.2012.04815.x 22702842

[30] Chen Y Tang W Liu C Lin J Wang Y Zhang S. miRNA-146a rs2910164 C>G polymorphism increased the risk of esophagogastric junction adenocarcinoma: a case-control study involving 2,740 participants. Cancer Manage Res. (2018) 10:1657–64. 10.2147/CMAR.S165921 29983589 PMC6025765

[31] Yin J Wang X Wei J Wang L Shi Y Zheng L. Interleukin 12B rs3212227 T > G polymorphism was associated with an increased risk of gastric cardiac adenocarcinoma in a Chinese population. Dis Esophagus (2015) 28:291–8. 10.1111/dote.12189 24529168

[32] Bland JM Altman DG. Multiple significance tests: the Bonferroni method. BMJ (1995) 310:170. 10.1136/bmj.310.6973.170 7833759 PMC2548561

[33] Lesack K Naugler C. An open-source software program for performing Bonferroni and related corrections for multiple comparisons. J Pathol Inform. (2011) 2:52. 10.4103/2153-3539.91130 22276243 PMC3263024

[34] Elrod HA Sun SY. PPARgamma and Apoptosis in Cancer. PPAR Res. (2008) 2008:704165. 10.1155/2008/704165 18615184 PMC2442903

[35] Girnun GD Smith WM Drori S Sarraf P Mueller E Eng C. APC-dependent suppression of colon carcinogenesis by PPARgamma. Proc Natl Acad Sci USA. (2002) 99:13771–6. 10.1073/pnas.162480299 12370429 PMC129773

[36] Sarraf P Mueller E Jones D King FJ DeAngelo DJ Partridge JB. Differentiation and reversal of malignant changes in colon cancer through PPARgamma. Nat Med. (1998) 4:1046–52. 9734398 10.1038/2030

[37] Tontonoz P Spiegelman BM. Fat and beyond: the diverse biology of PPARgamma. Annu Rev Biochem. (2008) 77:289–312. 10.1146/annurev.biochem.77.061307.091829 18518822

[38] Puigserver P Wu Z Park CW Graves R Wright M Spiegelman BM. A cold-inducible coactivator of nuclear receptors linked to adaptive thermogenesis. Cell (1998) 92:829–39. 10.1016/S0092-8674(00)81410-5 9529258

[39] Lauby-Secretan B Scoccianti C Loomis D Grosse Y Bianchini F Straif K. Body fatness and cancer–viewpoint of the IARC working group. N Engl J Med. (2016) 375:794–8. 10.1056/NEJMsr1606602 27557308 PMC6754861

[40] Grygiel-Gorniak B Kaczmarek E Mosor M Przyslawski J Bogacz A. Genetic background, adipocytokines, and metabolic disorders in postmenopausal overweight and obese women. Biochem Genet. (2016) 54:636–52. 10.1007/s10528-016-9743-z 27246401 PMC5018036

[41] Lv X Zhang L Sun J Cai Z Gu Q Zhang R. Interaction between peroxisome proliferator-activated receptor gamma polymorphism and obesity on type 2 diabetes in a Chinese Han population. Diabetol Metabol Syndrome (2017) 9:7. 10.1186/s13098-017-0205-5 28123453 PMC5248486

[42] Lu Y Ye X Cao Y Li Q Yu X Cheng J. Genetic variants in peroxisome proliferator-activated receptor-gamma and retinoid X receptor-alpha gene and type 2 diabetes risk: a case-control study of a Chinese Han population. Diabetes Technol Therap. (2011) 13:157–64. 10.1089/dia.2010.0122 21284483

[43] Du J Shi H Lu Y Du W Cao Y Li Q. Tagging single nucleotide polymorphisms in the PPAR-gamma and RXR-alpha gene and type 2 diabetes risk: a case-control study of a Chinese Han population. J Biomed Res. (2011) 25:33–41. 10.1016/S1674-8301(11)60004-3 23554669 PMC3596674

[44] Wang P Wang Q Yin Y Yang Z Li W Liang D. Association between peroxisome proliferator-activated receptor gamma gene polymorphisms and atherosclerotic diseases: a meta-analysis of case-control studies. J Atheroscler Thromb. (2015) 22:912–25. 10.5551/jat.26138 25832497

[45] Doecke JD Zhao ZZ Stark MS Green AC Hayward NK Montgomery GW. Single nucleotide polymorphisms in obesity-related genes and the risk of esophageal cancers. Cancer Epidemiol Biomarkers Prev. (2008) 17:1007–12. 10.1158/1055-9965.EPI-08-0023 18398047

[46] Zhou XP Smith WM Gimm O Mueller E Gao X Sarraf P. Over-representation of PPARgamma sequence variants in sporadic cases of glioblastoma multiforme: preliminary evidence for common low penetrance modifiers for brain tumour risk in the general population. J Med Genet. (2000) 37:410–4. 10.1136/jmg.37.6.410 10851250 PMC1734615

