# Supplementary material for: PPARG rs3856806 C>T Polymorphism Increased the Risk of Colorectal Cancer: A Case-Control Study in Eastern Chinese Han Population
Source: Front Oncol. 2019 Feb 19;9:63. doi: 10.3389/fonc.2019.00063 (PMC6389672; doi:10.3389/fonc.2019.00063)
Supplement: Supplementary file 1 [file Table_1.docx]

**Table S1** Logistic regression analyses of association of *PPARG* rs3856806 C>T polymorphisms and risk of CRC (Bootstrap Sample Trail)

| Genotype | Cases  (n=1,003) | |  | Controls  (n=1,303) | | Crude OR  (95%CI) | *P* | Adjusted OR ^a^  (95%CI) | *P* |
| --- | --- | --- | --- | --- | --- | --- | --- | --- | --- |
|  | n | % |  | n | % |  |  |  |  |
| *PPARG* rs3856806 C>T |  |  |  |  |  |  |  |  |  |
| CC | 549 | 54.74 |  | 785 | 60.25 | 1.00 |  | 1.00 |  |
| CT | 361 | 35.99 |  | 451 | 34.61 | 1.11(0.93-1.32) | 0.262 | 1.12(0.94-1.34) | 0.214 |
| TT | 70 | 6.98 |  | 62 | 4.76 | **1.56(1.09-2.23)** | **0.015** | **1.54(1.07-2.22)** | **0.020** |
| CT+TT | 431 | 42.97 |  | 513 | 39.37 | **1.20(1.02-1.42)** | **0.033** | **1.21(1.02-1.44)** | **0.027** |
| CC+CT | 910 | 90.73 |  | 1,236 | 94.86 | 1.00 |  | 1.00 |  |
| TT | 70 | 6.98 |  | 62 | 4.76 | **1.53(1.08-2.18)** | **0.017** | **1.51(1.05-2.16)** | **0.025** |
| T allele | 501 | 24.98 |  | 575 | 22.06 |  |  |  |  |

^a^Adjusted for age, sex, smoking status, alcohol use and BMI status.

Bold values are statistically significant (*P*<0.05)
